# Thermal biology of the sub-polar–temperate estuarine crab *Hemigrapsus crenulatus* (Crustacea: Decapoda: Varunidae)

**DOI:** 10.1242/bio.013516

**Published:** 2016-02-15

**Authors:** Juan P. Cumillaf, Johnny Blanc, Kurt Paschke, Paulina Gebauer, Fernando Díaz, Denisse Re, María E. Chimal, Jorge Vásquez, Carlos Rosas

**Affiliations:** 1 Instituto de Acuicultura, Universidad Austral de Chile, Casilla 1327, Puerto Montt, Chile; 2 Centro-IMAR, Universidad de Los Lagos, Casilla 557, Puerto Montt, Chile; 3 Laboratorio de Ecofisiologıá de Organismos Acuáticos, Departamento de Biotecnologıá Marina, Centro de Investigación Cientıf́ica y de Educación Superior de Ensenada (CICESE), Carretera Ensenada-Tijuana # 3918, Ensenada 22860, Baja California, México; 4 Unidad Multidisciplinaria de Docencia e Investigación, Facultad de Ciencias UNAM, Puerto de abrigo s/nSisal, Yucatán 97355, México

**Keywords:** *Hemigrapsus crenulatus*, Preferred temperature, Critical thermal limits, Oxygen consumption, Haemolymph protein

## Abstract

Optimum temperatures can be measured through aerobic scope, preferred temperatures or growth. A complete thermal window, including optimum, transition (Pejus) and critical temperatures (CT), can be described if preferred temperatures and CT are defined. The crustacean *Hemigrapsus crenulatus* was used as a model species to evaluate the effect of acclimation temperature on: (i) thermal preference and width of thermal window, (ii) respiratory metabolism, and (iii) haemolymph proteins. Dependant on acclimation temperature, preferred temperature was between 11.8°C and 25.2°C while CT was found between a minimum of 2.7°C (CT_min_) and a maximum of 35.9°C (CT_max_). These data and data from tropical and temperate crustaceans were compared to examine the association between environmental temperature and thermal tolerance. Temperate species have a CT_max_ limit around 35°C that corresponded with the low CT_max_ limit of tropical species (34–36°C). Tropical species showed a CT_min_ limit around 9°C similar to the maximum CT_min_ of temperate species (5–6°C). The maximum CT_min_ of deep sea species that occur in cold environments (2.5°C) matched the low CT_min_ values (3.2°C) of temperate species. Results also indicate that the energy required to activate the enzyme complex (Ei) involved in respiratory metabolism of ectotherms changes along the latitudinal gradient of temperature.

## Abstract (precis)

**Summary:** The preferred temperature of the intertidal temperate crab *Hemigrapsus crenulatus* was determined to be between 12°C and 25°C. Oceanic warming would trap this species between tropical and polar temperatures, causing increasing population stress with unknown ecological consequences.

## INTRODUCTION

Temperature is an important factor that affects the patterns of geographic distribution and abundance of ectothermic organisms (Terblanche et al., 2011). The thermal tolerance boundaries in organisms are determined by the combination of morphological, physiological and biochemical features, such as the sensitivity of structural and enzymatic proteins (Somero, 2004; Tepolt and Somero, 2014). Temperature has a direct effect on the velocity of biochemical reactions and the channelling of energy to maintain homeostasis and, as a consequence, the physiological status of aquatic organisms (Zi-Ming et al., 2013). Environmental temperature influences a variety of organismal processes in ectotherms, including growth, reproduction and survival. Organisms have thus evolved a variety of strategies to regulate body temperature. Those strategies fall into three broad categories: (1) behavioural, (2) physiological, and (3) morphological regulation. Behavioural regulation by means of movement and body reorientation are effective to avoid potentially damaging body temperatures, and are commonly used by a variety of ectotherms (Gaitan-Espitia et al., 2013). Understanding on the distribution and abundance of organisms in relation to temperature requires information on their physiological capabilities that are reflected in their thermal window width (Tepolt and Somero, 2014); this information acquires special relevance in a climate change scenario.

In the more classical definition critical thermal limits (CT) have been defined as “the thermal point at which locomotory activity becomes disorganised and the animal loses its ability to escape from conditions that will promptly lead to its death” (Lutterschmidt and Hutchison, 1997b). In this definition, the authors suggested that a constant heat rate should be used “allowing deep body temperature to follow ambient test temperatures without a significant time lag”. Loss of the righting response and muscular spasms were identified as the final point of critical temperature. This dynamic method is widely used to define the limits of the thermal tolerance zone of many ectothermic species (Pörtner, 2001; Terblanche et al., 2011; Madeira et al., 2014; Ern et al., 2015; Noyola et al., 2016). More recently, Pörtner (2010) and Sokolova et al. (2012) proposed that thermal window of ectotherms is defined by the aerobic scope where optimum temperature interval is located where aerobic scope is maximum and CT is the extreme limit of thermal tolerance where the aerobic scope may be near to zero. The temperature interval between CT and optimum was termed Pejus and was identified as a transition temperature interval where protection mechanisms against radical oxygen species are activated. In an attempt to standardize dynamic and oxygen-limited and capacity-limited thermal tolerance (OCLTT) hypothesis it is possible to consider that CT limits obtained by both methods are reflecting in different form the same thermal limit. At the end, wherever the method used thermal window attributes provide insight into ectothermic ecology and have been used to quantify the thermal niche of several fresh and marine species (Bennett and Beitinger, 1997; Eme and Bennet, 2009; Noyola et al., 2013).

Different methods have been used to determine the thermal optimal zone in aquatic organisms, e.g. measurements of oxygen consumption (Chen and Lai, 1993; Díaz et al., 2013; Fry, 1947; Magozzi and Calosi, 2014; Manush et al., 2004; Nilsson et al., 2009; Rummer et al., 2014), cardiac activity (Braby and Somero, 2006; Oellermann et al., 2012; Tepolt and Somero, 2014) and thermal preference (Angilletta et al., 2002; Beitinger and Fitzpatrick, 1979; Ern et al., 2015; Lewis and Ayers, 2014; Noyola et al., 2013; Padilla-Ramírez et al., 2015; Paschke et al., 2013; Reiser et al., 2014a; Reynolds and Casterlin, 1979). Thermal preference is widely used to establish optimal conditions given that it confirms the hypothesis of co-adaptation. For instance, preferred temperatures may correspond to optimal temperatures because maximum physiological performance occurs at those conditions (Angilletta et al., 2002). A polygon can be constructed given that optimal and Pejus zones can be obtained through the aerobic scope and through the preferred temperatures and CT limits (Reynolds and Casterlin, 1979; Sokolova et al., 2012). In addition, the area of thermal window can be calculated from preferred temperatures and from the CT_max_ and CT_min_ limits of animals acclimated at different temperatures. The area of thermal window (reported as °C^2^) has been used as a comparative index of thermal tolerance between species (Eme and Bennet, 2009) and allows defining the amplitude of maximum and minimum tolerance zones in a particular species. In terms of adaptation, the thermal window appears to be useful for understanding thermal capacities, how organisms have evolved to colonise specific environments, or how they will tolerate environmental changes (Pörtner, 2002, 2006, 2010; Pörtner and Farrell, 2008; Pörtner et al., 2005).

Abbreviations

CT: critical temperature (°C)
CT_max_: critical thermal maximum (°C)
CT_min_: critical thermal minimum (°C)
Ei: activation energy (eV K^−1^)
OCLTT: oxygen-limited and capacity-limited thermal tolerance hypothesis
FP: final *preferendum*
TTI: thermal tolerance interval
ww: wet weight
Q_10_: respiratory coefficient
*V*_O_2__: volume of oxygen consumed (O_2_ h^−1^ g^−1^)
ENSO: El Niño Southern Oscillation
TTA: thermal tolerance area (°C^2^)
eV: electron volt
K: temperature in Kelvin
ΔH: enthalpy

Species that inhabit the intertidal and estuarine zones have developed physiological, morphological and behavioural adaptations to complete all or part of their life cycle in this fluctuating environment. Between physiological mechanisms, which were identified in response to temperature increase, are those that allow *Carcinus maenas* (Linnaeus) regulate their respiratory metabolism to stabilise oxygen consumption. Such regulation occurs via compensatory mechanisms directed to delay hypoxemia and increase the amount of oxygen bound to haemocyanin (Giomi and Pörtner, 2013). In *Macrobrachium rosenbergii* (De Man) and *Penaeus monodon* (Fabricius) was observed that this mechanism is linked with an increment of cardiac work at high temperatures which allows them to maintain metabolic scope at temperatures below the critical temperatures (Ern et al., 2014, 2015).

The OCLTT concept defines the temperature window of a species as a function of the extent and maintenance of its aerobic scope, as required for energy allocation to growth, behaviour and immune functions (Pörtner, 2010). If the metabolism and nutritional condition of crustaceans are modulated by their life habits, e.g. sedentary (*Callapa spp;* Weber), medium active (*Callinectes similis* Rathbun) and active (*Farfantepenaeus aztecus;* Ives, and *Litopenaeus setiferus* Linnaeus) (Pascual et al., 2003; Sánchez et al., 1991), then metabolic rate can be related with the life habits and in consequence with thermal tolerance of Crustacea.

The euryhaline crab *Hemigrapsus crenulatus* (H. Milne-Edwards) is a common species in the mid to high intertidal zone of estuarine southern Chile. This Varunidae crab is widely distributed along the coast of Chile, from Africa in the north (18°S) to the Estrecho de Magallanes in the south (53°S) (Díaz-Jaramillo et al., 2013), and in New Zealand from Parengarenga Harbour (34°31′S) to Stewart Island (47°02′S) (Davie and Ng, 2007; McGaw, 2003). While temperate species are expected to have limited acclimation capacities as they live in thermal stable environments, *H. crenulatus* is a species with tropical origin inhabiting estuarine environments in a temperate environment where they experience wide temperature fluctuations (6°C to 24°C). In this context, the present study was divided in three parts: One was directed to evaluate the effect of acclimation temperature in: (i) the thermoregulatory behaviour (thermal preference and width of thermal window) and (ii) respiratory metabolism of adult *H. crenulatus* in an attempt to obtain a better understanding of its thermal biology, temperature tolerance limits and its potential performance as colonizing species that could be favoured by gradual increments of temperature in temperate zones of South of American Pacific Ocean.

Second part of the study was done taking into account that a relationship between OCLTT concept and life habitat could exist, we compared the effects of temperature on metabolic rate, tolerance and width of thermal windows of several crustacean species inhabiting tropical, temperate and cold environments in an attempt to provide insights into how the life habitat of different crustacean species are related to their thermal tolerance.

In third place and according to Pörtner (2006), a wide thermal window in sub polar organisms with lower activation energy is characteristic of those organisms because of the unstable and cold environment where they live. Using data of *H. crenulatus* and data from literature we tested this hypothesis

## RESULTS

### Thermal window of *H. crenulatus*

**Fig. 1.**
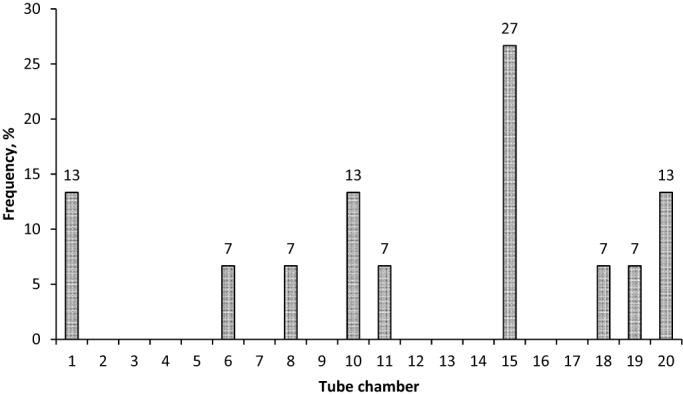
**Frequency distribution of *Hemigrapsus crenulatus* in the tube chambers at** **12°C.** Percentage of animals that occupied a particular section (chamber) of tube when the experimental tube was maintained at a constant temperature of 12°C (Control experiment without the thermal gradient, *n*=15).

**Fig. 2.**
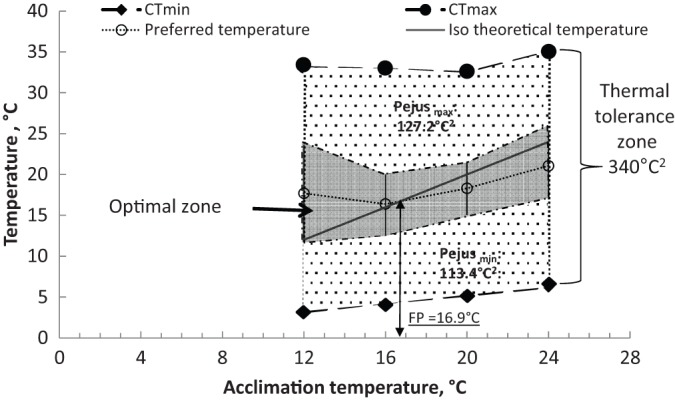
**Preferred temperatures, CT_min_ and CT_max_, and thermal window for *Hemigrapsus crenulatus* acclimated at different temperatures.** Iso-theoretical temperature indicates when the temperature selected by crabs and the acclimation temperature are similar. With this line and preferred temperatures it is possible to calculate final *preferendum* (arrow). Area for maximum and minimum thermal Pejus (Pejus_max_ and Pejus_min_, respectively) were calculated considering that CT indicates the critical threshold where aerobic scope is close to zero (Sokolova et al., 2012). The optimal zone was identified with the preferred temperatures. Values are mean±s.d.; *n*=13, 16, 12 and 12 for crabs acclimated at 12, 16, 20 and 24°C, respectively; *n*=9 for CT_max_ and *n*=9 for CT_min_ for crabs acclimated at each experimental temperature.

There were no significant differences in the average weight of the animals between different groups corresponding to the experimental treatments (*P*=0.187). Crabs placed in the tube with sea water at 12°C (control experiment) did not select any particular chamber. A relatively high number of crabs (27%) selected the chamber 15, other crabs (13%) selected chambers 1, 10 and 20. The rest of crabs were randomly distributed along the tube (Fig. 1). The preferred temperatures of crabs at different acclimation temperatures treatments were 17.7±5.9, 16.4±3.9, 18.3±3.3 and 21.1±4.1°C, respectively. There were no significant differences between preferred temperatures (*P*>0.05; Fig. 2; Table 1). The final *preferendum* value of 16.9°C was obtained graphically (Fig. 2). The interval between preferred temperatures at each acclimation temperature treatment was considered the optimal zone in the thermal window of *H. crenulatus* (Fig. 2).

**Table 1.**
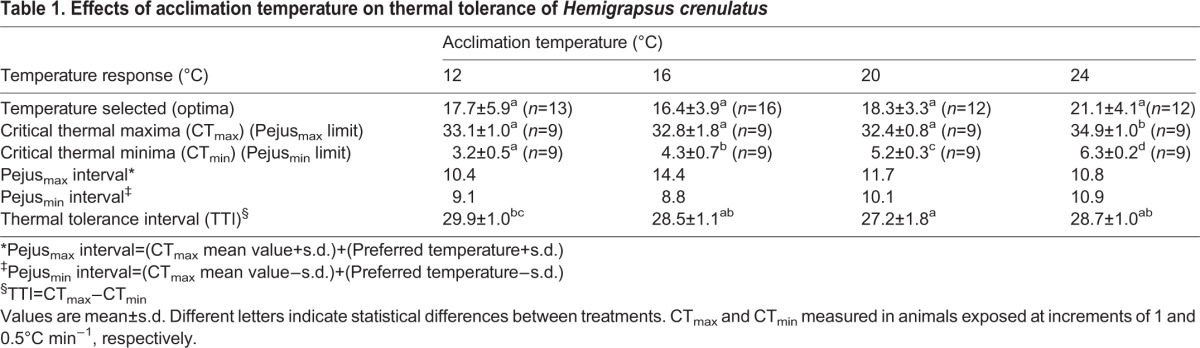
**Effects of acclimation temperature on thermal tolerance of *Hemigrapsus crenulatus***

The acclimation temperature affected the CT_max_ (*P*<0.05), and varied from 33.1±0.84 to 34.94±1.00°C in organisms acclimated at 20 and 24°C, respectively. The highest CT_max_ value (34.94°C) obtained from crabs acclimated at 24°C was significantly higher than CT_max_ values from crabs acclimated at 12, 16 and 20°C (mean value of 32.6°C; *P*<0.001; Fig. 2). A linear increment of CT_min_ was observed according to acclimation temperature with low values for animals maintained at 12°C (3.21±0.53°C) and high values for crabs acclimated at 24°C (6.28±0.24°C; *P*<0.001; Fig. 2). *Hemigrapsus crenulatus* exhibited an area of thermal window of 340°C^2^ that was divided in three zones (Fig. 2). Two zones identified the Pejus area in the thermal window: Pejus_max_ (127.2°C^2^) and Pejus_min_ (113.4°C^2^). The area calculated for optimal zone into the thermal window of *H. crenulatus* was 99.4°C^2^ (Fig. 2).

The calculated thermal tolerance interval fluctuated between 27.19±0.36°C and 29.86±0.46°C for *H. crenulatus* crabs acclimated at 20°C and 12°C, respectively. Crabs acclimated at 16°C and 24°C had similar temperature intervals between 28.50±1.23°C and 28.67±0.47°C, respectively (Table 1).

**Fig. 3.**
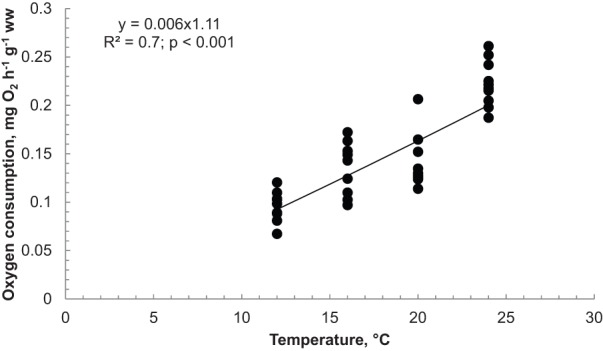
**Effect of different acclimation temperatures on the oxygen consumption rate of *Hemigrapsus crenulatus*.** A liner relationship was observed between oxygen consumption (mg O_2_ h^−1^ g^−1^ ww) and increased acclimation temperature. *n=*10 per treatment.

Oxygen consumption rate (*V*_O_2__) was affected by acclimation temperature, with mean values of 0.095±0.015, 0.14±0.026, 0.13±0.015, and 0.24±0.06 mg O_2_ h^−1^ g^−1^ wet weight (ww) for acclimation temperatures of 12, 16, 20 and 24°C, respectively (*P*<0.001). A linear relationship between oxygen consumption and temperature was obtained from these data (Fig. 3). The range of temperature coefficient (Q_10_) was higher (4.6) in crabs acclimated between 20 and 24°C compared with the Q10 (1.20) of crabs acclimated between 16–20°C.

**Fig. 4.**
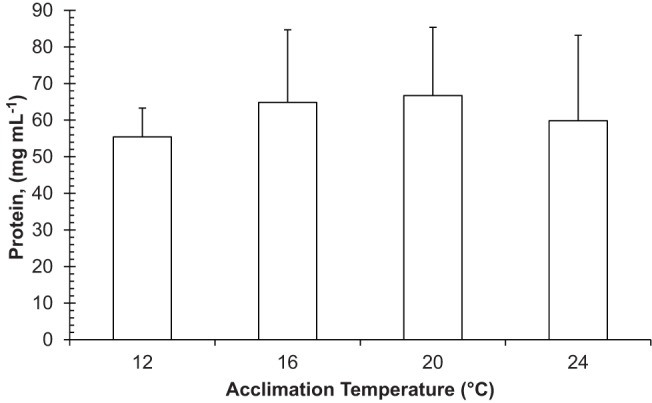
**Effect of acclimation temperature on plasma soluble protein of *Hemigrapsus crenulatus*.** ANOVA analysis indicated that haemolymph protein levels were not affected by acclimation temperature. Values are mean+s.d. *n=*10 crabs per treatment.

Haemolymph protein levels were not affected by acclimation temperatures (*P*>0.05) and mean values of 1.37±0.40 mmol l^−1^ Hc and 61.71±18.21 mg ml^−1^ protein were calculated (Fig. 4).

## DISCUSSION

This study described the complete thermal window for *H. crenulatus* including optimal, transition (Pejus) and critical limits. The preferred temperature and critical temperatures were placed as equivalents of optimal and critical threshold of the OCLTT concepts (Pörtner, 2010). The standardization of these terms was made taking in consideration that preferred temperature matches maximum growth and performance in several ectotherms (Angilletta et al., 2002; Lewis and Ayers, 2014), and that critical temperature is the limit of the temperature tolerance zone where the time of survival is limited (Ern et al., 2015; Pörtner, 2010; Sokolova et al., 2012).

The best performance of *H. crenulatus* occurred between 12°C and 25°C and its final preferred temperature was 16.9°C, which are good indicators of the thermal environmental requirements for this species. In accordance with this finding, the organisms examined were physiologically stable within this range of temperature, where minimum stress was observed and their physiological functions were optimised (Angilletta et al., 2002). Depending on acclimation temperature, we hypothesise that the optimal zone for maximum aerobic scope of *H. crenulatus* occurs between 12°C and 25°C. Moreover, we observed that animals acclimated at 12–24°C did not modify their haemolymph protein contents. Crabs at those temperatures may thus have mechanisms to transport sufficient oxygen that, at mitochondrial level, may regulate tolerance to high temperatures (Giomi and Pörtner, 2013; Pörtner, 2002).

The thermal tolerance of other marine crabs has also been described, e.g. *Petrolistes cinctipes* (Randall), CT_max_=32.6°C and 33.9°C; *P. manimaculis* (Glasell), CT_max_=28.9°C and 31.0°C; and *P. cabrilloi* (Glasell) CT_max_=32.6°C and 34.9°C, acclimated at 8–13°C and 18°C, respectively (Stillman, 2002). *Hemigrapsus nudus* (Dana), CT_max_=31.1°C and 33.6°C acclimated at 10 and 16°C (McGaw, 2003); and *C. maenas* (Linnaeus), CT_max_=34.2°C to 36.4°C acclimated at 5°C and 25°C (Tepolt and Somero, 2014). We propose that CT_max_ for *H. crenulatus* corresponds to the critical threshold temperature where the scope of metabolic activity is zero. Under these conditions animals lost their righting response and showed muscular spasms that reflected their deficit of energy that was necessary to scape. A collapse of heart rate and gill ventilation rate in *M. rosembergii, P. monodon* and *Astacus astacus* (Linnaeus) occurs in CT, indicating a failure of the mechanisms to transport enough oxygen to mitochondria (Ern et al., 2014, 2015). Therefore, in critical temperatures organisms can stay alive for short time where animals enter a phase of reparation acclimation (Pörtner, 2010; Ern et al., 2015). Animals acclimated at 24°C had higher CT_max_ values (35°C) compared with other treatments (mean value of 32.8°C). Hence, crabs acclimated at 24°C may be near their limit of tolerance to temperature. We observed that it was not possible to acclimate *H. crenulatus* at 28°C, where crabs died after 4 h. Hence, intervals between 12°C and 24°C may correspond to the functional acclimation temperatures where the maximum performance can be observed (Pörtner, 2010). Our findings suggest that *H. crenulatus* may have a limited ability to increase its thermal tolerance above the higher limits of acclimation and could be at risk due to the ocean warming scenarios projected by the IPCC (2013).

As was observed in intertidal fish (Madeira et al., 2014) in *H. crenulatus* the Pejus interval were considered to reflect temperature interval where acclimation in protection occurs (Pörtner, 2010; Sokolova et al., 2012). The Pejus_max_ interval was higher than the Pejus_min_ interval for all acclimation temperatures; this finding suggests that *H. crenulatus* has a wider tolerance to higher temperatures than to lower temperatures. For instance, the Pejus_max_ area was 13.8°C^2^ higher than the Pejus_min_. At those ranges of acclimation temperature (12°C to 24°C), *H. crenulatus* is likely to use adaptive mechanisms to support its tolerance to high temperatures rather than to low temperatures, but with lethal limits around 28°C.

The estuarine habitat where *H. crenulatus* occurs reaches temperatures around 24°C during an average summer. Given that temperatures higher than 24°C may be sub-lethal for this species, crabs are likely to move southwards in a warming scenario as they track cooler temperatures. It is expected that under warming scenarios the habitat of *H. crenulatus* will be restricted, which is likely to have ecological consequences. For instance, the habitat of this species may be delimited by warm temperatures at the North and by the cool circumpolar current at the South. To test that hypothesis we can use El Niño Southern Oscillation (ENSO) events that provoke temperature increments around 2°C and result in changes in the structure of populations of crustaceans and molluscs that occur in the coastal zone of the Peruvian Province (Paredes et al., 2004). In thermal terms the ENSO event is a cyclic and temporal warming that occurs due to changes of atmospheric pressures in the Pacific Ocean (Trenberth and Hoar, 1996). During ENSO, sea surface temperatures can reach more than 30°C and modify ecological characteristics of the coastal zone. Colonization is a common response of tropical crustaceans and molluscs along the Peruvian Province during ENSO events favouring species that tolerates high temperatures over species that are adapted to sub-tropical environments (Paredes et al., 2004). In this context ENSO promotes thermal refuges that allow tropical species to temporally colonize relatively cooler ecosystems when the sea temperature increases. Eventually, colonizing individuals decrease in number with the end of ENSO conditions (e.g. when temperatures decrease). Those changes on ecological structure of crustaceans and molluscs during ENSO help to us to hypothesise that during warming, *H. crenulatus* population of the South of Chile could be forced to move to more cooler coastal environments, as now is occurring with many species of crustaceans in Peruvian Province during temporal warming provoked by ENSO events.

**Fig. 5.**
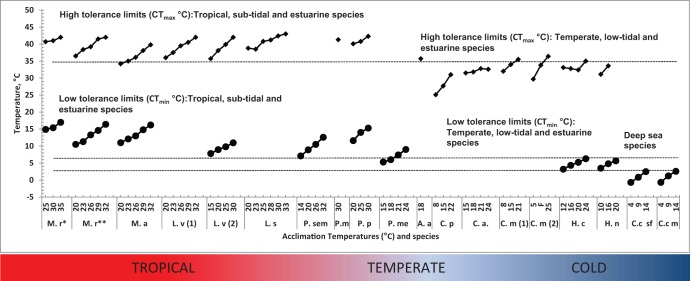
**CT_max_ and CT_min_ of different crustaceans that occur in tropical, temperate and deep sea cold habitats, including *H. crenulatus*.** Dotted lines indicate the frontiers that separate low and high limits of tolerance at high and low temperatures, depending on the habitat of the species. Temperate species have a CT_max_ limit around 35°C that corresponded with the low CT_max_ limit of tropical species (34–36°C). Tropical species showed a CT_min_ limit around 9°C similar to the maximum CT_min_ of temperate species (5–6°C). The maximum CT_min_ of deep sea species (2.5°C) that occur in cold environments matched the low CT_min_ values (3.2°C) of temperate species. M.r*, *Macrobrachium rosenbergii* (Manush et al., 2004); M.r**, *M. rosenbergii* (Díaz Herrera et al., 1998); M.a, *M. acanthurus* (Díaz et al., 2002); L.v(1), *Litopenaeus vannamei* (González et al., 2010); L.v(2), *L. vannamei* (Kumlu et al., 2010); L.s, *L. stylirostris* (Re et al., 2006); P.sem, *Penaeus semisulcatus* (Kir and Kumlu, 2008); P.m, *Penaeus monodon* (Ern et al., 2015); P.p, *Portunus pelagicus* (Qari and Aljarari, 2014); P.me, *Penaeus merguiensis* (Hoang et al., 2002); A.a, *Astacus astacus* (Ern et al., 2015); C.p, *Cancer pagurus,* and C.m(1), *Carcinus maenas* (Cuculescu et al., 1998); C.a., *Cancer antenarius* (Padilla-Ramírez et al., 2015); C.m (2), *C. maenas*, including field data from California (F) (Tepolt and Somero, 2014); H.c, *Hemigrapsus crenulatus* (this study); H.n, *H. nudus* (McGaw, 2003); C.c sf., *Crangon crangon* spent females and C.c m., *C. crangon* males (Reiser et al., 2014a,b).

Comparisons of temperature tolerance of *H. crenulatus* and other tropical, sub-polar or deep sea crustaceans (Fig. 5) allowed detecting well-defined temperature limits among crustaceans. Analysing the superior limit of tolerance (CT_max_) it is possible to see that tropical animals acclimated at low temperatures showed CT_max_ values around 37°C, similar to the highest CT_max_ observed in temperate animals acclimated at high temperatures (Fig. 5). Similarly, the maximum CT_min_ (2.5°C) values obtained in the deep sea shrimp *Crangon crangon* (males and spent females; Reiser et al., 2014a,b) acclimated at the highest temperature (14°C) were relatively similar to the minimum CT_min_ of *H. crenulatus* and *H. nudus* and other temperate species acclimated at low temperatures, indicating that critical thermal maximum limits measured as in the present study can be used to identify the thermal adaptation of the species at their specific habitat. (Cuculescu et al., 1998; Ern et al., 2015; McGaw, 2003; Noyola et al., 2016; Padilla-Ramírez et al., 2015; Tepolt and Somero, 2014).

When CT_min_ limits are analysed a similar thermal frontier appears between tropical-temperate and temperate-cold crustacean species (Fig. 5). Tropical shrimps acclimated at low temperatures showed CT_min_ values between 5.3–7.8°C while temperate species acclimated at high temperatures had CT_min_ values between 5.8–6.3°C. In addition, CT_min_ values (i.e. 3°C) for *C. crangon* acclimated at high temperature (i.e. 14°C; Reiser et al., 2014a,b) was comparable with the lowest CT_min_ values (i.e. 3.2°C and 3.5°C) for *H. crenulatus* and *H. nudus* acclimated at low temperatures suggesting that the low limit for tropical animals may be the high limit for temperate animals, as well as the temperature limit for deep sea species that inhabit cold environments (Fig. 5). Low CT_min_ values of −0.68°C observed in deep sea organisms, such as adult, male and spent female *C. crangon* (Reiser et al., 2014a,b), suggest that this species is well adapted to live in cool environments, where physiological traits may allow colonization of polar environments under ocean warming scenarios.

**Fig. 6.**
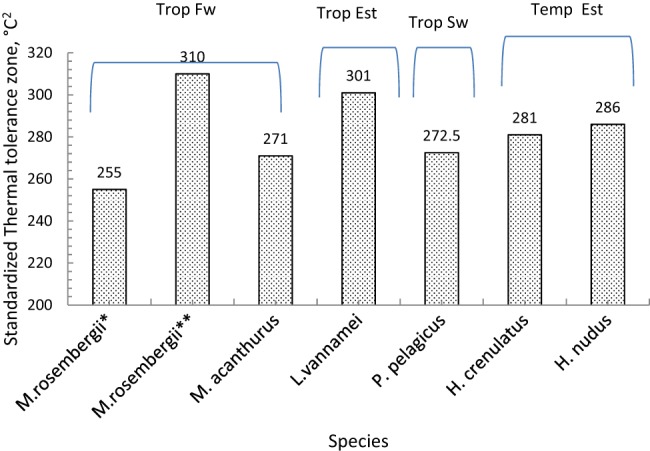
**Thermal tolerance zone (area) standardized to 10°C intervals of acclimation temperatures for prawns, shrimps and crabs with different geographic distributions.** An analysis of standardized thermal tolerance areas (TTAs) of a range of crustaceans revealed considerable differences in TTA between tropical and temperate marine and estuarine species. Trop Fw, tropical and fresh water habitat; Trop Sw, tropical and sea water habitat; Trop Est, tropical and estuarine distribution; Temp Est, temperate and estuarine distribution. Species analysed: Macrobrachium rosembergii* (Manush et al., 2004); M rosembergii** (Díaz Herrera et al., 1998); *M. acanthurus* (Díaz et al., 2002); *Litopenaeus vannamei* (González et al., 2010); *Portunus pelagicus* (Qari and Aljarari, 2014); *Hemigrapsus crenulatus* (this study); *H. nudus* (McGaw, 2003).

To determine the width of the thermal window of *H. crenulatus* and other crustaceans, we calculated thermal tolerance areas (TTA: °C^2^) for *M. rosenbergii* (two strains)*, M. acanthurus, L. vannamei, P. pelagicus, H. crenulatus* and *H. nudus* acclimated at 10°C (Fig. 6). Considerable differences in TTA were observed between tropical and temperate marine and estuarine species and *H. crenulatus*. For instance, the tropical shrimp *L. vannamei* and *M. rosenbergii* Mexican strain have a higher tolerance zone (301 and 310°C^2^, respectively) compared with the tropical *P. pelagicus* (272.5°C^2^), and the temperate *H. crenulatus* (281°C^2^) and *H. nudus* (286°C^2^) suggesting that temperate species could be more sensible to warming. In this sense Noyola et al. (2016) showed that tropical crabs *Callinectes similis* (Rathbun) and *Libinia dubia* (Milne-Edwards) have thermal window (336 and 304°C^2^, respectively) similar to those of tropical shrimp and prawns confirming that idea that tropical species of crustaceans have higher thermal windows that temperate species. In a recent study Deutsch et al. (2015) showed that due to increments of temperature and dissolved oxygen by warming, tropical species will tend to migrate to the poles, searching better environmental conditions. The metabolic index calculated in these studies suggests that due to species migration marine environments will be compressed. In such scenario tropical species with wide thermal windows will colonize the new habitats changing ecology and fisheries.

Routine oxygen consumption measured in *H. crenulatus* showed energy demands of basal metabolism and routine metabolism of unfed and non-stressed animals (Clarke, 2004; Clarke and Fraser, 2004). The evolutionary trade-off hypothesis proposes that “temperature dictates the overall slope of the relationship between resting metabolic rate and temperature in organisms adapted to live at different temperatures through a combination of energetic trade-off and evolutionary temperature adaptations” (Clarke, 2004). In this hypothesis, the metabolic rate represents the energetic cost of evolutionary adaptation to a particular temperature and can be used to identify differences between species with similar life habits.

**Fig. 7.**
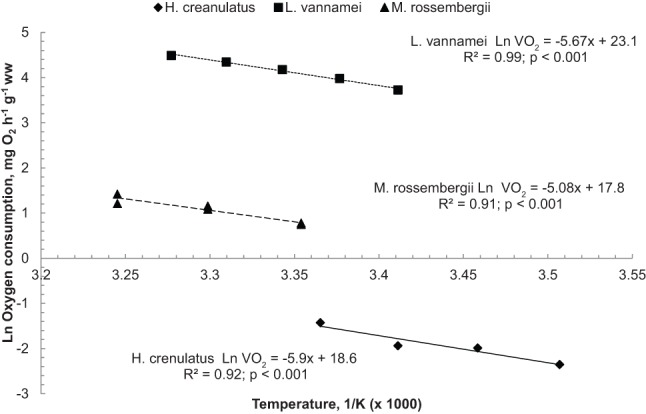
**Effect of temperature on oxygen consumption in tropical and sub-polar–temperate crustaceans.** An Arrhenius plot of temperature vs oxygen consumption reveals that energy required to activate the enzyme complex (Ei) involved in respiratory metabolism is higher in tropical (*L. vannamei,* González et al., 2010; *M. rosenbergii,* Manush et al., 2004) than in sub-polar–temperate (*H. crenulatus*, this study) species.

The Arrhenius plot provides an excellent description of the relationship between metabolism and temperature because it is based in the first principles of thermodynamics (Clarke and Johnston, 1999). Therefore, an Arrhenius plot was constructed to explore the effects of temperature on the activation energy of species with high (*L. vannamei;* González et al., 2010), medium (*M. rosenbergii;* Manush et al., 2004) and low activity (*H. crenulatus,* this study) (Fig. 7). *H. crenulatus* had the greatest slope in the plot followed by *L. vannamei* and *M. rosenbergii*, suggesting that the energy required to activate the enzyme complex (Ei) involved in respiratory metabolism is higher in species which are not active (*H. crenulatus*, Ei=−0.51 eV K^−1^) than in active species (*L. vannamei*, Ei=−0.49 eV K^−1^; and *M. rosenbergii*, Ei=−0.44 eV K^−1^). Our results suggest that the hypothesis that explains the differences in Ei between cold and temperate species extends to tropical and temperate species as well (Pörtner, 2006). This hypothesis states that there is an association between temperature dependence of mitochondrial proton leakage and its consequences in the free enthalpy of activation (ΔH). In stenotherms, a high Ei mirrors the high ΔH and therefore a low metabolism. Without enough aerobic energy, stenothermal species have narrower thermal windows than eurythermal species. Stenothermal organisms have higher Ei, lower metabolism and narrower thermal windows than eurythermal organisms. A comparison between *H. crenulatus* and tropical species showed a similar trend; tropical crustaceans have a wider thermal window than *H. crenulatus* suggesting that the relationship between activation energy, proton leakage and metabolism could be present along the latitudinal gradient of temperature. Clarke and Johnston (1999) found similar results where the resting oxygen consumption increases with environmental temperatures; which suggests that an increment in aerobic scope can be associated to wider thermal windows due to environmental demands (Pörtner, 2006).

The coefficient of temperature (Q_10_) of *H. crenulatus* was lower at 16°C to 20°C (Q_10_=1.20), indicating that in this range of temperature organisms were better acclimated to maintain homeostasis. Similar results were observed in several aquatic species like fish (Beitinger and Bennet, 2000; Beitinger et al., 2000), shrimp (Díaz et al., 2013; González et al., 2010) and octopus (Noyola et al., 2013).

Our findings suggest that the mechanisms involved in the adaptation of *H. crenulatus* to low temperature are related to its capacity to synthesize protein in a wide range of experimental temperatures, in addition to enzymatic and metabolic adaptations for metabolic rate regulation. Haemolymph protein concentrations were not affected by the wide range of experimental temperatures where *H. crenulatus* was acclimated. In this sense, this species appears to be able to respond to environmental variations at temperate in the sub-polar estuaries which they inhabit.

In the present study we proposed equivalences of thermal nomenclatures to standardize terms on the thermal windows for aquatic ectotherms. Critical temperatures, i.e. maximum and minimum temperatures, were proposed as critical threshold temperatures for the OCLTT model. Pejus was defined as the difference between the interval of selected temperatures and critical temperatures, which allowed estimating the thermal window for *H. crenulatus*. Further studies should verify if there is an association between aerobic scope, preferred temperatures and biochemical mechanisms in the temperature tolerance of this species. Based on our results we propose a hypothesis to explain why active animals have higher tolerance windows than less active animals. This idea was further supported by differences in Ei observed between *H. crenulatus* and tropical Decapods. However, this is just a mere hypothesis that requires further research.

## MATERIALS AND METHODS

### Acclimation of experimental animals

A total of 165 crabs *H. crenulatus* were collected in the intertidal and sub-tidal Cariquilda River, in Southern Chile (41°37′6.74″S, 73 35′33.76″W). Animals were immediately transported to the laboratory, weighted (9.13±2.59 g wet weight), measured (23.5±2.12 mm carapace width) and randomly sorted into four experimental groups (40–44 individuals each). Each experimental group was maintained for 21 days at a specific experimental temperature (12, 16, 20 and 24°C) in 60-litre flow-through seawater tanks. To control the temperature, each tank was equipped with an Atman electronic heater of 300 W (Shangai, China) connected to digital thermostats (Danfoss model EKC102A, Santiago, Chile). The temperature was verified with a portable computer WTW Cond 330i conductivity-meter connected to a digital thermometer (Cooper, DPP400W model, Alabama, USA). The animals were kept in a 12:12 h light:dark photoperiod and fed *ad libitum* with bivalve molluscs. Females and males were selected during the intermoult period and none of the specimens were ovigerous females. No mortalities were recorded during the acclimation period or during the experiment. The effects of temperature acclimation on oxygen consumption, and total protein were evaluated in the blood of a sub-group of crabs (10 individuals per acclimation temperature). All animal experimental protocols comply with Chilean welfare guidelines.

### Thermal preference

**Fig. 8.**
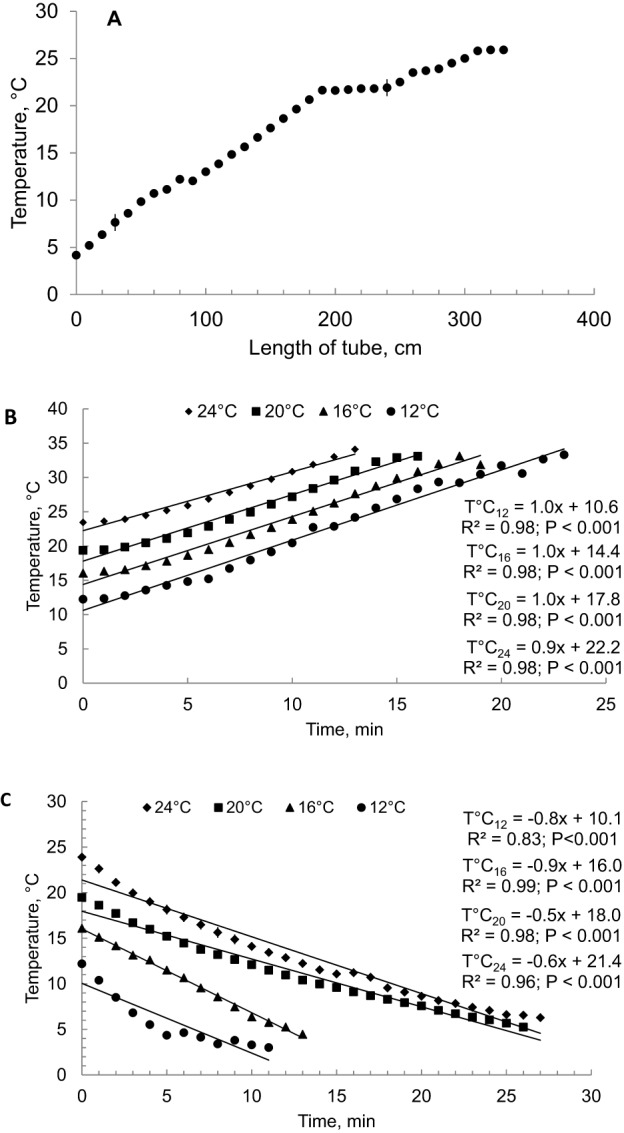
**Establishment of** **critical thermal maximum (CT_max_) and critical thermal minimum (CT_min_) for *Hemigrapsus crenulatus*.** Temperatures in the tube used to examine the thermal preference (A) and temperature changes when CT_max_ (B) and CT_min_ (C) were evaluated in *H. crenulatus* acclimated at 12, 16, 20 and 24°C. Temperatures obtained during each measurement are showed as mean±s.d.; *n*=13,16, 12, and 12 for crabs acclimated at 12, 16, 20 and 24°C, respectively; *n*=9 for CT_max_ and *n*=9 for CT_min_ for crabs acclimated at each experimental temperature.

The thermal preference was determined for each crab using the acute method with a horizontal gradient of temperature (Paschke et al., 2013; Reynolds and Casterlin, 1979). The apparatus consisted of a PVC pipe (330 cm long and 16 cm diameter) with 21 virtual sections of 15 cm each. The system was maintained in a temperature-controlled room at 8°C and the depth of the water column was 9 cm. A temperature gradient was formed by placing two 300 W Atman heaters (Shangai, China) at one end and frozen seawater at the other. The gradient had a temperature interval of 4.1–27°C (Fig. 8A). In each virtual segment, a tube diffuser was placed along the gradient to provide very gentle aeration, to maintain a concentration of dissolved oxygen close to saturation, and to avoid stratification in the water column. The temperature was measured in each virtual segment with a digital thermometer.

A control experiment was performed to evaluate the distribution of crabs in the tube when a homogenous temperature of 12°C was maintained. Fifteen crabs were used in this control experiment (Fig. 1). In the temperature gradient experiments, three tagged crabs were simultaneously introduced at the virtual section that corresponded to their acclimation temperature (12, 16, 20 or 24°C). The location of the organisms and the temperature of each section were recorded every 10 min for a total of 60 min. Although preference observations were done every 10 min, temperature preferred was only registered at the end of the time of exposition into the tube. Consequentially we have only 13, 16, 12 and 12 crabs observed at 12, 16, 20 and 24°C, respectively. The final *preferendum* (FP) was determined graphically using the intersection of the organisms' preferred temperature at each temperature acclimation with the iso-theoretical temperature equality line. This line is constructed based on the theoretical temperature selection of the crab being equal to the acclimation temperature.

### Determination of critical thermal maximum (CT_max_) and minimum (CT_min_)

A 40-litre aquarium was used as a thermo-regulated bath containing three experimental cylindrical chambers of 1 litre. Each chamber had a digital thermometer (Cooper, DPP400W model, Alabama, USA), and an air-stone was used to maintain oxygen saturation and to avoid thermal stratification in the water column. For CT_max_, the aquarium had a 1500 W immersion heater (Atman, Shangai, China) attached to an air-stone that maintained a uniform temperature in the bath. For CT_min_, the bath was conditioned as a cold chamber, using an ice bed saturated with NaCl powder. Determination of CT_max_ and CT_min_ was performed between 09:00 and 14:00 h. Nine *H. crenulatus* animals from each experimental temperature were used for CT_max_ and for CT_min_, respectively. The heating and cooling rates in the aquaria were ranged approximately between 1 and −0.5°C min^−1^, respectively (Fig. 8B,C). We used the temperature increase and decrease rates because they allow the muscle and nervous system temperatures change with the change of sea water temperature (Cuculescu et al., 1998; Lutterschmidt and Hutchison, 1997a,b). The behavioural stress response of *H. crenulatus* to change in temperature was visually monitored. CT_max_ and CT_min_ endpoints were identified when crabs showed loss of the righting response, i.e. when crabs were on their back and could not recover an upright posture within one minute (Paschke et al., 2013). When the crabs reached this point, they were returned to the acclimation temperature. The animals were used only once and monitored during 96 h for recovery; none of the animals died during this experiment. A thermal tolerance interval (TTI) was calculated for each acclimation temperature as the difference between CT_max_ and CT_min_ mean values (Paschke et al., 2013).

Thermal window amplitude was calculated when CT_max_ and CT_min_ data were integrated with the preferred temperatures in the same figure. The preferred temperatures (mean±s.d.) of animals acclimated at experimental temperatures were those found in the optimal zone, whereas CT_max_ and CT_min_ were the Pejus_max_ and Pejus_min_ limits. The area covered by each zone was calculated considering 12°C in the *x* axis (12°C to 24°C of acclimation temperatures) and its corresponding interval of temperatures of each zone (optimal, Pejus_max_ and Pejus_min_). The thermal window amplitude for *H. crenulatus* was the sum of all areas.

### Oxygen consumption

Oxygen uptake was quantified individually in closed plastic respirometers (*n*=10). The animals from each experimental temperature were carefully introduced in the respirometric 1-litre chambers 18 h before initiating the measurements to avoid interference by stress (Martínez-Palacios et al., 1996). Animals were incubated in UV-sterilised and filtered seawater (31±0.5 practical units of salinity; filtered at 1 µm) at the corresponding acclimation temperature. Observations were made between 9:00 and 14:00 h. The respirometric chambers remained closed for 30 min to prevent the dissolved oxygen from decreasing less than 30% of its concentration and becoming a source of stress (Stern et al., 1984). Before closing the chambers, a water sample was taken to quantify the initial concentration of oxygen. After 30 min, another sample was taken to measure the final concentration of dissolved oxygen. The sea water was also renewed in the respirometric chamber to increase the concentration of oxygen close to the saturation level. Two repetitions were performed, in a 2 h interval, with 10 crabs from each experimental temperature. The oxygen concentration was measured before and after using an optical sensor connected to oxygen-measuring equipment (Microx PreSens; TX3, Regensburg Germany) already calibrated with air (100% saturation) and 5% sodium sulphite solution (0% saturation). A control chamber without crabs was used to determine the potential oxygen consumption by bacterial activity in the chambers. The oxygen consumption (mg O_2_ h^−1^ g^−1^ ww) of crabs was calculated from the difference between the initial and final concentrations of oxygen at each acclimation temperature.

The thermal coefficient for the metabolic rate of crab (Q_10_) represents the organism's sensitivity to temperature variation. The calculation of Q_10_ took into account animals acclimated at 12–16°C, 16–20°C and 20–24°C and was derived by the equation (Eckert et al., 1989):

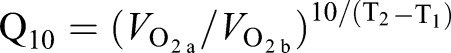
where *V*_O_2a__ and *V*_O_2b__ are the metabolic rates at temperatures T_2_ and T_1_, respectively.

### Total protein in the blood

Total protein levels were examined in the blood of 10 crabs from each acclimation temperature. Haemolymph samples were extracted from the arthrodial membrane at the base of the fourth pair of walking legs using a disposable and pre-cooled 1-ml syringe (Pascual et al., 2003). A 100-µl haemolymph sample was obtained and quickly transferred into a pre-cooled 1.5-ml Eppendorf tube. A 10-μl haemolymph sub-sample was used to estimate total protein with a protein assay kit (Lowry et al., 1951) (BioRad DC Protein Assay, Sao Paulo, Brazil) modified for a microplate reader. Haemolymph samples were read at 750 nm and the calibration curve was constructed using bovine serum albumin as the standard solution (1.4 mg ml^−1^).

### Statistical analysis

Levene and Kolmogorov–Smirnov tests were performed to examine assumptions of homogeneity of variance and normality. One-way analysis of variance (ANOVA) was used to evaluate the effect of acclimation temperature on living weight, total protein in the blood of crabs, thermal preference, CT_max_ and CT_min_. Differences among groups were assessed using the Kruskal–Wallis test (oxygen consumption, CT_max_) when the data did not meet the assumptions of homogeneity of variance and normality. Multiple comparisons were examined with Student–Newman–Keuls test. The *t*-test was used to examine differences between the experimental temperatures and acclimation temperatures. Differences were significant at *P*<0.05 (Zar, 1999).
